# Gene families as soft cliques with backbones: *Amborella *contrasted with other flowering plants

**DOI:** 10.1186/1471-2164-15-S6-S8

**Published:** 2014-10-17

**Authors:** Chunfang Zheng, Alexey Kononenko, Jim Leebens-Mack, Eric Lyons, David Sankoff

**Affiliations:** 1 Department of Mathematics and Statistics, University of Ottawa, 585 King Edward Avenue, Ottawa, Canada, K1N 6N5; 2 Department of Plant Biology, University of Georgia, Athens, GA 30602-7271, USA; 3 The School of Plant Sciences, University of Arizona, Tucson, AZ 85721 USA

**Keywords:** gene families, clustering, *S*-plex, angiosperms, *Amborella trichopeda*

## Abstract

**Background:**

Chaining is a major problem in constructing gene families.

**Results:**

We define a new kind of cluster on graphs with strong and weak edges: soft cliques with backbones (SCWiB). This differs from other definitions in how it controls the "chaining effect", by ensuring clusters satisfy a tolerant edge density criterion that takes into account cluster size. We implement algorithms for decomposing a graph of similarities into SCWiBs. We compare examples of output from SCWiB and the Markov Cluster Algorithm (MCL), and also compare some curated *Arabidopsis thaliana *gene families with the results of automatic clustering. We apply our method to 44 published angiosperm genomes with annotation, and discover that *Amborella trichopoda *is distinct from all the others in having substantially and systematically smaller proportions of moderate- and large-size gene families.

**Conclusions:**

We offer several possible evolutionary explanations for this result.

## Background

The automatic detection of clusters of vertices in a graph is practiced in diverse fields from image recognition to social networks, and is widely used in computational biology for the study of gene families. Conceptually, a gene family is a set of genes, in one genome or several, that includes all descendants of a single gene in some ancestral organism (i.e., homologous genes), and excludes genes descended from other ancestral genes (i.e., non-homologous genes). Operationally, lacking the historical data to identify a gene family in these terms, it is standard practice to construct gene families on the basis of DNA or protein sequence similarities. The assumption is that genes in the same family will retain much more sequence similarity than unrelated genes, though this is more of a general tendency than a strict rule. The genes belonging to a particular gene family may be identified with the vertices of a graph, which has edges between pairs of genes exceeding a threshold similarity score.

In the present work, we will focus on gene families within a single genome. We set aside data on syntenic correspondences between orthologs as well as functional evidence relating genes, despite their usefulness in many contexts, in order to achieve the first of our two goals - the identification of the conceptual and methodological problems in the purely graph-theoretical approach, and the framing of a proposal to deal with them.

In plants, the creation, expansion and attrition of gene families through mechanisms of gene duplication, notably tandem duplication and, more spectacularly, whole genome doubling, allows rapid adaptation of populations to a broad range of niches. This motivates the second of our two goals in this paper - a comprehensive survey of gene family sizes in 44 published angiosperm genomes.

In the first part of this paper, we review some of the desiderata of clustering methods in graphs, and define a new kind of cluster: *soft cliques with backbones *(SCWiB). Though similar in some respects to methods based on Minimum Spanning Tree, SCWiB clustering controls the "chaining effect" characteristic of many such methods, by requiring that clusters satisfy a tolerant edge density criterion that takes into account cluster size. We present an exact algorithm based on the SCWiB concept that can handle moderate amounts of data, and that can be converted into a heuristic for realistic genomes.

We then compare SCWiB results with the output of MCL [1,2], a method which is one of the most widely used for inferring gene families. We also see how the SCWiB families compare with the curated gene families of *Arabidopsis thaliana *[3].

Finally we apply our algorithm to 44 published angiosperm genome sequences. We compare the distribution of gene family sizes, and find similar patterns are displayed in the large majority of cases. We find, however, that the earliest branching angiosperm, *Amborella trichopoda *has a distinctly different pattern, with relatively few moderate- or large-size families.

## Results

Creating gene families on the basis of similarities is essentially a kind of clustering. Well-known clustering methods like *k*-means [4], hierarchical methods, e.g., single- link [5], average-link [6] and complete- link, spatial methods, e.g., PCA and self-organizing maps [7], and graph-based methods, e.g., minimum spanning trees [8] and cliques, have all been used. These all have advantages and disadvantages, depending on the context. In our study of gene families, we wanted to avoid methods that produce large, inhomogeneous, families by "chaining", such as single-link, on the one hand, and methods that are overly biased towards smaller or equal-sized families, like clique or complete-link, on the other.

Chaining is a major problem in constructing gene families, largely due to the multiple domain structure of many proteins. Some domains recur in many different families, with the result that both conceptually and operationally, there are no longer strict boundaries between families. This problem has been treated in most depth by Joseph and Durand [9,10]. Methods that construct clusters by adding objects to that cluster with an element closest to them, without accounting for the rest of the cluster, like single-link or minimum spanning tree are particularly prone to chaining and, in some applications, like recognition of objects in satellite imagery, this may be desirable [11,12]. However, in the context of constructing gene families, this amounts to the inclusion of non-homologous genes within the same family, something to be avoided in evolutionary analyses

### Gene families as soft cliques with backbones

To ensure that a gene family is determined by strong similarities connecting each of its members,

• we set a high similarity threshold *U *and require that a cluster be connected, in the graph theoretical sense, solely in terms of similarities exceeding *U*. By itself this is similar to other graph theory criteria, and like them it is susceptible to chaining, for meaningful values of *U *or, alternatively, to very small clusters, if *U *is too high. To control for chaining

• we also set a less stringent threshold *W *, and require that the elements in the cluster form a clique, or almost form a clique, in terms of similarities exceeding *W*. We cannot require that the cluster forms a full clique, since this is too stringent for high values of *W*, and is not restrictive enough for low values. A way of relaxing the clique criterion is

• to require the similarities in a cluster to form an *S*-plex [13], where *S *= *sN+1*, the number of elements in the cluster being *N *, and 0 <*s *< 1 is a constant. In an *S*-plex, every element is of degree at least *N *− *S*.

Each cluster is thus validated on two levels, as a set of strongly connected elements, at level *U*, that is not built by chaining, due to the *S*-plex condition at level *W*.

#### Exact algorithm

The algorithm generates all possible SCWiBs in a graph. The output can then be post-processed to find a compatible subset of these to satisfy any one of several criteria.

    **Algorithm SCWiB**

    **Parameters: **thresholds 0 <*W *<*U *< 1 and tolerance coefficient 0 <*s *< 1

    **Input: **graph *G*(*V, E*) with edge-weights *w*(·)

    **Output: **the list  L of the possible SCWiBs in *G*.

    **Steps:**

        define *E_U_*= {*e *∈ *E|U *≤ *w*(*e*)}, *E_W_*= {*e *∈ *E|W *≤ *w*(*e*) <*U*}

        order vertices by increasing degree

        while there are more vertices

            select the first vertex *v *from the ordered list of vertices

            call **ListgeneFamily**(*v, G, s, L*_1_)

            store *L*_1_ results in  L.

            remove *v *from *G*

    **Algorithm ListGeneFamily(*v, G, s, L*_1_)**

    **Input: **vertex *v*, graph *G*(*V, E*), *E *= *E_U _*∪ *EW*

    **Output: **the list *L*_1 _of the all the SCWiBs in *G *that contain *v*

    **Steps:**

        let *d *be the degree of vertex *v*

        maximum size *m *of SCWiB that can contain *v *is $m=\frac{d}{s}+1$

        create a queue *Q*, insert the subgraph with only one vertex *v *into *Q*

        while there are more subgraphs in *Q*

            pop out the first subgraph *sg *and store it into *L*_1_

            if *|sg| *<*m*,

                for each vertex *u *∈ *V* (and ∉ *sg*) joined with an edge in *E_U _*to the last vertex of *sg*,

                check if the subgraph *G'*(*V', E'*) of *G *is a SCWiB cluster, (*V' *= vertices of *sg *∪ {*u*}. *E' *is the edge set induced by *V'*) if yes, insert *G' *into *Q*.

The SCWiB algorithm presented here shows how the clusters can be calculated naturally, despite two independent levels of control on cluster quality. This is an exact algorithm as it constructs all possible clusters and then picks the largest, the next disjoint largest and so on. It requires exponential time, since the number of possible clusters can be exponential. As displayed it is simple, but unnecessarily inefficient; the algorithm can be sped up enormously by reordering the vertices after a specified number of calls to the inner algorithm. It can also be made more efficient by temporarily postponing the construction of clusters that threaten to require excessive time, and by a number of other devices. For use on large genomes, it can be converted into a heuristic by replacing the exhaustive exploration of all search paths by a large enough sample of these paths.

#### Comparison with MCL

**Figure 1:**
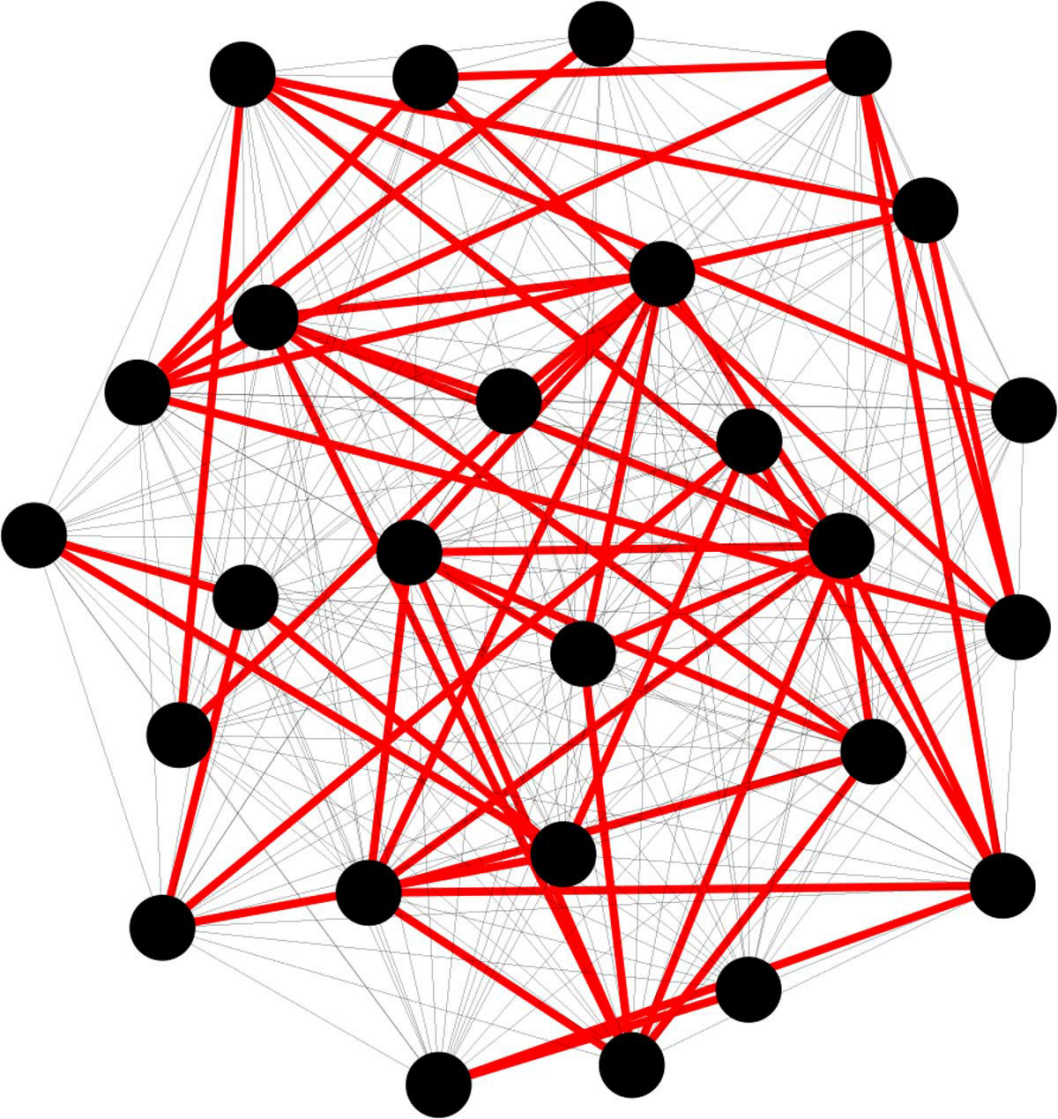
**SCWiB cluster containing part of the NAC transcription factor family **[60]. Dots represent genes. Red edges constitute the "backbone" with similarity greater than *U *, black edges indicate similarity greater than *W *, less than *U *.

Figure 1 shows an example of a SCWiB cluster emerging from an analysis of the *Arabidopsis thaliana *genome. It can be seen that although some of the genes are connected to the cluster by only one or two edges of similarity greater than *U *, any tendency to chaining is controlled by the *S*-plex condition at level *W *, with every vertex having a high degree within the cluster.

**Figure 2:**
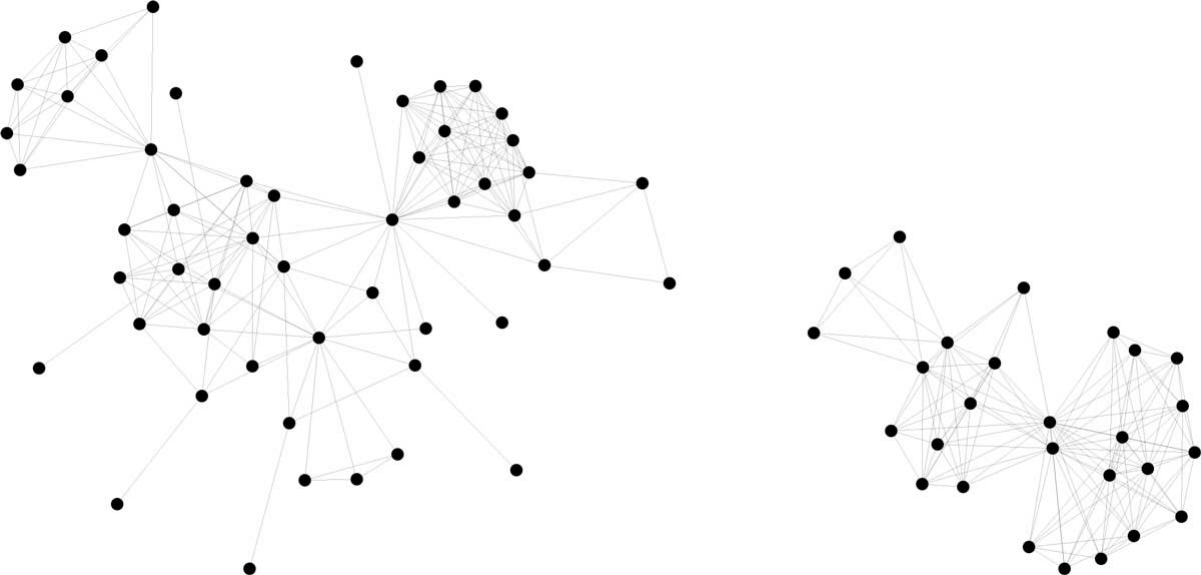
**Two MCL groups, inflation factor 1.6, showing chaining effect**.

MCL [1,2] is one of the most widely used methods for inferring gene families. Its basic principle is the iteration of a procedure that strengthens certain heavily weighted edges and weakens those with lesser weight. With appropriate parameter settings, MCL and SCWiB can produce very similar distributions of cluster sizes. The lack of any cluster quality criterion influencing the MCL process, however, results in many of its clusters, including some of the largest ones, having very few internal edges, as in Figure 2, while the SCWiB construction explicitly prohibits this.

#### Comparison with TAIR

The most comprehensive, though very incomplete, inventory of curated gene families for a plant pertains to the *Arabidopsis thaliana *genome [3]. This is a collection of gene families, found on the *Arabidopsis *Information Resource (TAIR) gene family page, contributed by individual scholars and groups, based largely on function within the cell as well as sequence similarities. It is not an attempt to partition the entire set of *Arabidopsis *genes into clusters, and there is no requirement that the families are disjoint. Furthermore, the functional groups are not intended to correspond perfectly with gene families as defined by common ancestry. Nonetheless, we compare these families with those produced by SCWiB. We find that many of the gene similarities in large functionally-determined families do not meet the SCWiB criteria, which therefore splits them into a number of subfamilies. The same holds for the comparison of the functional families with MCL clusters. This implies the limitations of purely similarity-based methods for gene family detection. Nevertheless, many functional families are in almost one-to-one correspondence with gene families determined by SCWiB.

In Figure 1, only part of the NAC transcription factor family is in the cluster; other parts are in other SCWiB clusters. This family has been diverging in the land plants long before the emergence of the angiosperms, so that different ancient NAC transcription factor subfamilies are not connected at the *U *= 70% level that we used. Of interest is that in an MCL analysis of this same data, with inflation factor fixed at 1.6 to achieve the same total number of gene families as SCWiB, this cluster is fragmented among five MCL families, none of them containing more than nine of the 26 genes.

#### The Angiosperm genomes

The emergence of new genes and new functions for existing genes is a major aspect of evolutionary divergence of species. In animals, especially the mammals, a key mechanism for such innovation is alternative splicing, which affects at least 50% of genes [14]. In plants, however, this phenomenon is thought to be much less important, impacting just 5-10% [15], while the creation, expansion and attrition of gene families through mechanisms of gene duplication, notably tandem duplication and, more spectacularly, whole genome doubling, may spur rapid adaptation of populations to a broad range of niches. We extracted all the data available on angiosperm genomes in the CoGe database [16,17]. We required genomes to be published, publicly available, and have associated structural gene annotations. The genomes included *Amborella*, soybean, *Brachypodium distachyon, Setaria*, peach, cassava, *Capsella rubella*, sorghum, eucalyptus, common bean, grapevine, cacao, banana, turnip, papaya, *Arabidopsis thaliana*, tomato, potato, *Arabidopsis lyrata, Leavenworthia alabamica, Sisymbrium irio, Aethionema arabicum*, strawberry, *Thellungiella parvula*, watermelon, sacred lotus, *Utricularia, Spirodela polyrhiza*, date palm, pigeonpea, sweet orange, poplar, rice, *Ricinus communis*, clementine, lotus, flax, maize, cucumber, kiwifruit, *Mimulus, Medicago*, pepper and *Eutrema parvulum *[18-59]. We could not exercise any control on the quality of the sequencing, the assembly, or the annotation, and we will discuss the possible consequences of this on our results in the Conclusions.

We used the SynMap tool in CoGe to run a comparison of each genome with itself in order to construct a complete set of gene duplicates. We disregarded syntenic context (pertinent only to WGD duplicates), by setting the minimum block length to 1. From the unfiltered results, we eliminated duplicates with similarities less than *W *= 0.6.

We decomposed the set of resulting set of duplicates into SCWiBs with parameters *U *= 0.7, *W *= 0.6, *s *= 0.25. We used a local optimization criterion, finding the largest possible SCWiB first, then re-applying the method on successively small graphs that result from removing the vertices in the previously generated clusters. It should be noted that SynMap, as we used it produces a large peak of duplicate genes with similarities from 60-64. This had little if any consequence for our results, since almost all of these duplicates would be eliminated by the *U *criterion, although they could provide support for the *S*-plex criterion.

Based on *U *= 0.7, our gene families would largely have origins within the angiosperms, or be subfamilies of ancient plant gene families diversifying within the angiosperms.

**Figure 3:**
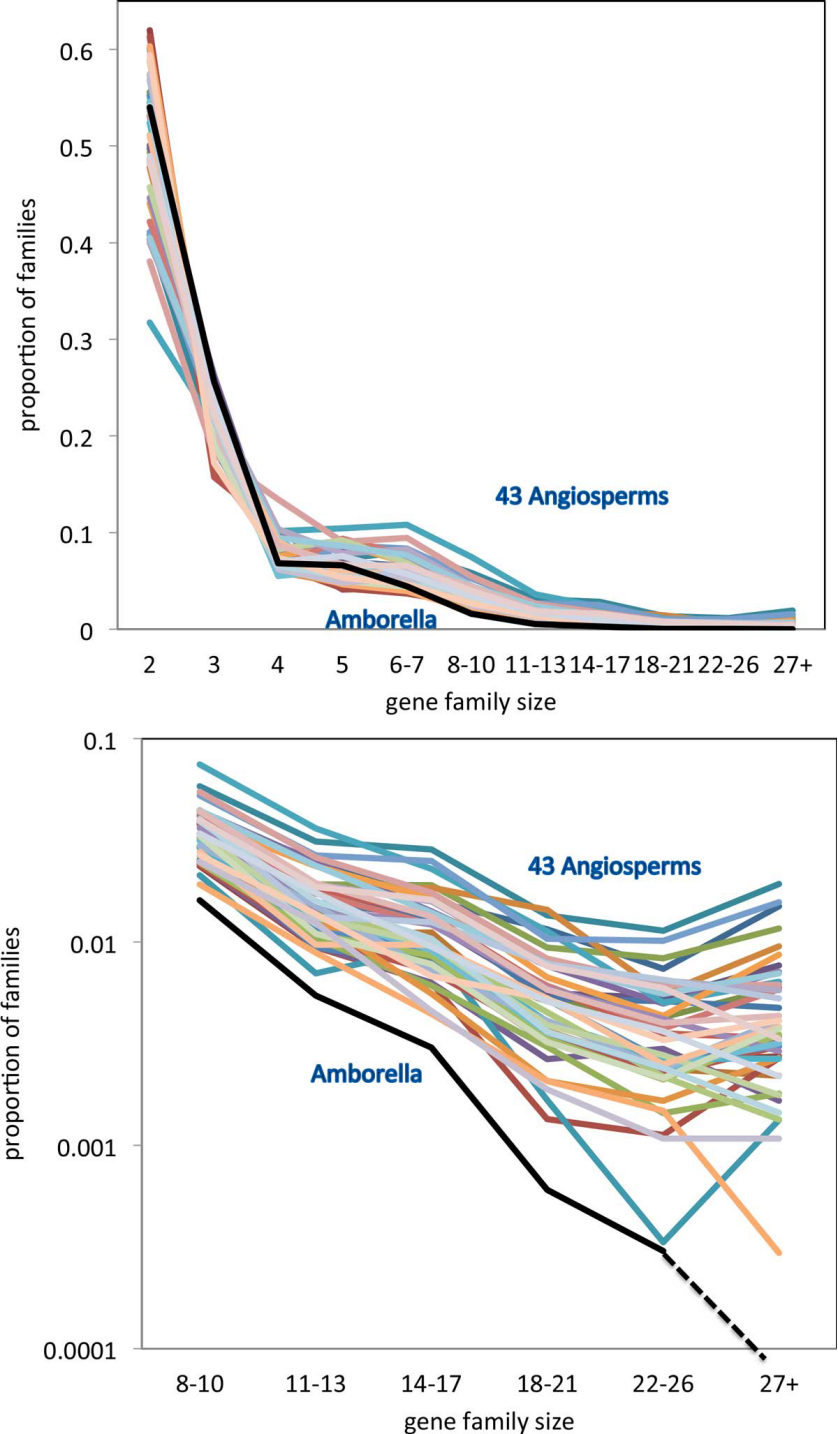
**Proportion of gene families of various sizes**. Singletons not included. Top: entire range of sizes. Bottom: moderate and large families. *U *= 0.7, *W *= 0.6, *s *= 0.25.

**Figure 4:**
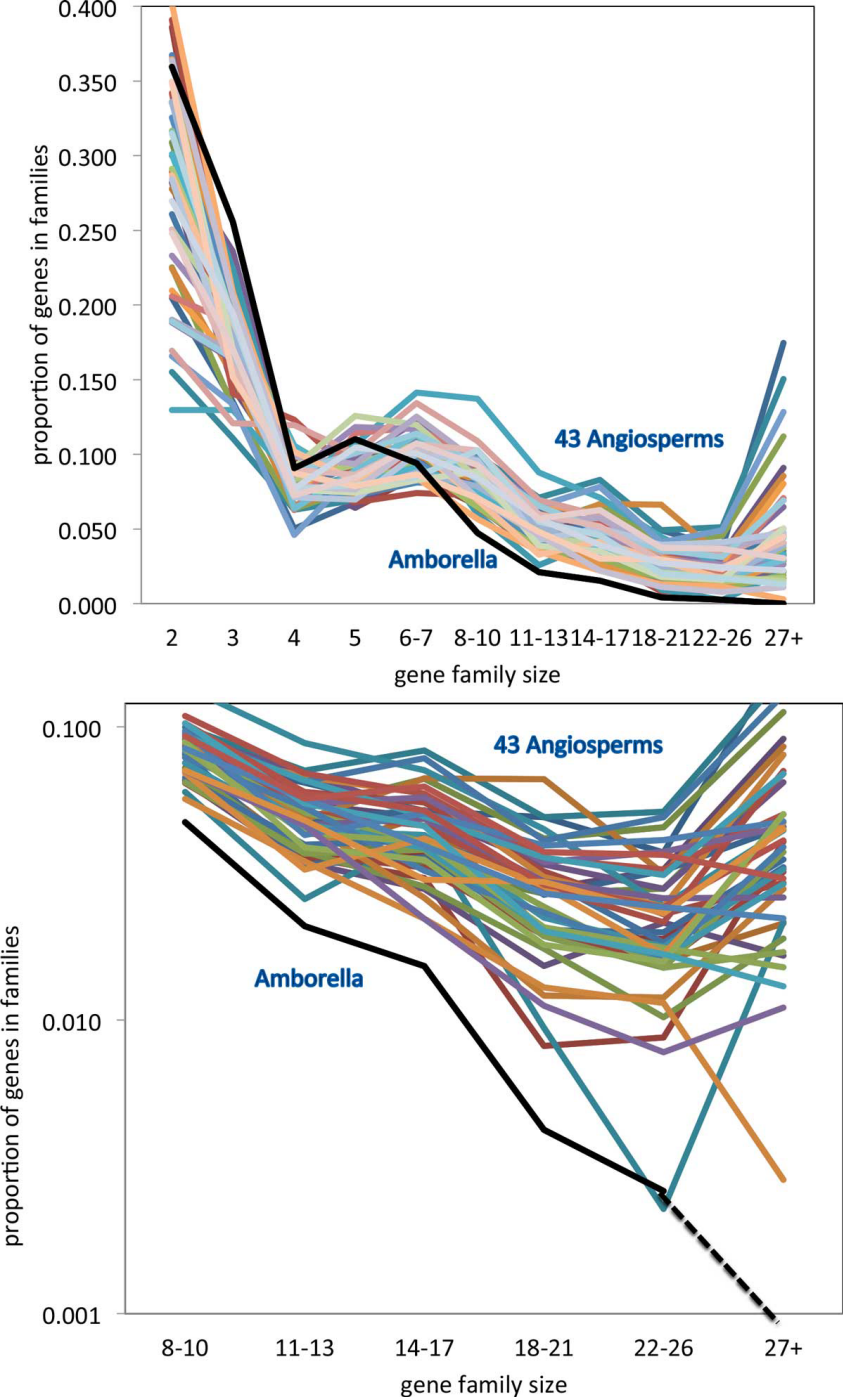
**Proportion of genes in families of various sizes**. Singletons not included. Top: entire range of sizes. Bottom: moderate and large families. *U *= 0.7, *W *= 0.6, *s *= 0.25.

Figure 3, displaying relative numbers of families of each size, and Figure 4, with the total number of genes in these families, show broadly similar gene family size distribution across the angiosperms, but also show a remarkable trend involving the *Amborella trchopoda *genome. Whether we measure it according to number of gene families of a given size, or according to the proportion of genes in gene families of a given size, *Amborella *has fewer gene families of moderate (starting at 8-10 members) or of large size (22-26, 27 or more), than any of the other genomes.

**Figure 5:**
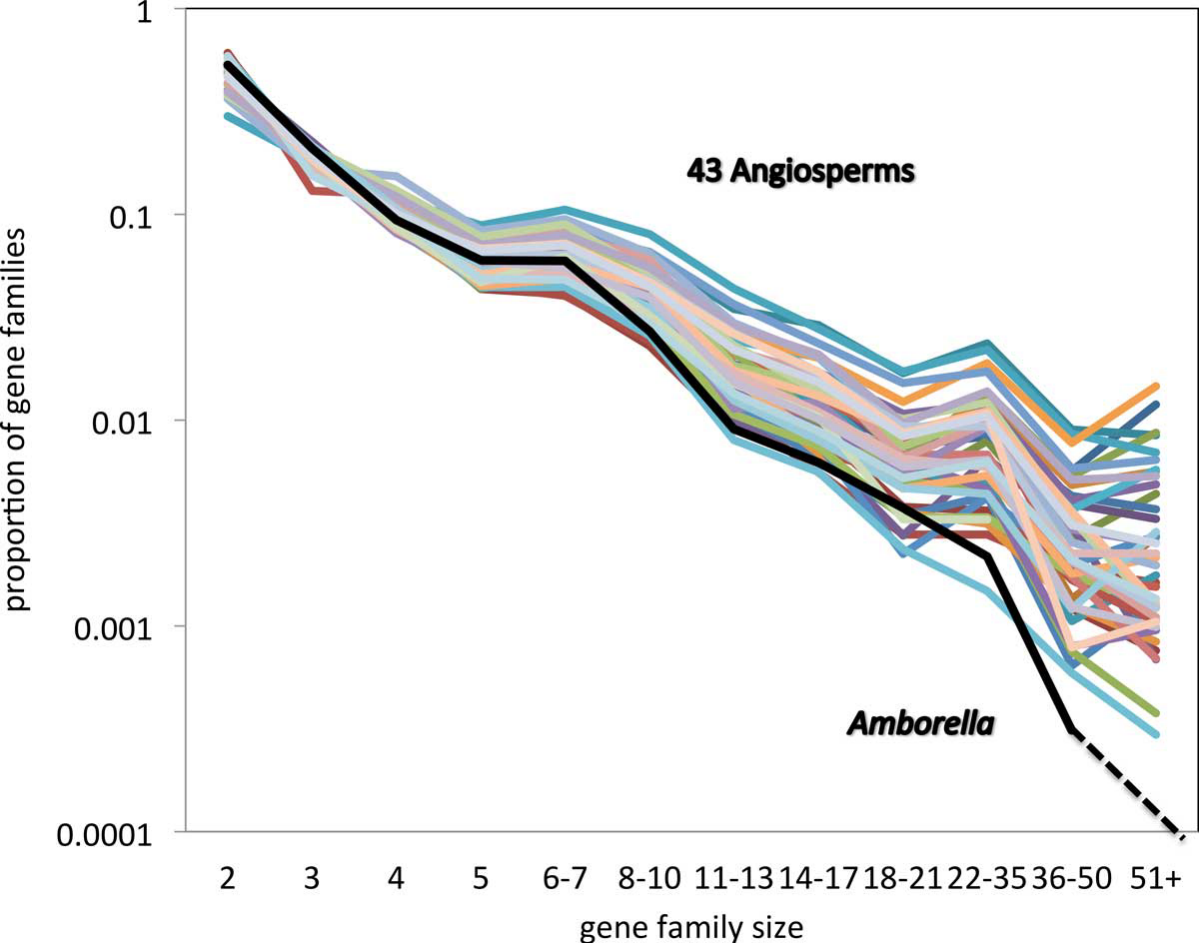
**Proportion of gene families of various sizes in MCL analysis**. Singletons not included. Inflation factor 1.6.

As a control, we carry out an experiment on the same set of gene pairs for each genome, but using the MCL method. Exactly the same genes are involved. To ensure that the number of gene families were comparable, we used an inflation factor of 1.6 for the MCL. Figure 5 shows that distribution of MCL family sizes is more spread out than in the SCWiB case in Figure 3. However, the anomalous lack of large gene families of *Amborella *still stands out. This pattern emerges clearly, although the distinction is not as clear as with SCWiB. Another genome, cucumber, also has small numbers of moderate-sized families.

## Discussion and conclusion

The parameters of SCWiB directly control the connectedness and density characteristics of the clusters; we can predict the results of changes in each one. This contrasts with program parameters like the inflation factor in MCL, whose effects are largely indirect and unpredictable at the level of individual clusters. Although SCWiB involves three numerical factors, they enter into the algorithm in a simultaneous way to assure both connectedness and density. SCWiB clusters can also be generated by heuristics derived from generic search strategies such as branch-and-bound, and we have implemented this as a check on results from the **algorithm SCWiB**-derived heuristics.

The remarkably distinct pattern apparent in the *Amborella *distribution of gene family sizes will have to be validated in future studies. Most immediate is the role of the specific parameter values for *U, W *and *s*. Does the pattern hold up when one or more of these are shifted? Preliminary results, not shown here, are positive: increasing *s *from 0.25 to 0.35 increases the number of larger gene families (size *>*26) for all the genomes, but the distinction between *Amborella *(which only sees one family achieving a size of 30) and the other 43, is amplified. A systematic answer to this question will require considerable computing time to experiment with different values of *U *and *W *, but without any change in methodology. Another question is whether the pattern we observe is somehow dependent on the SCWiB definition, We have shown that the MCL method, which differs from SCWiB in almost every way possible, reproduces the distinct pattern of *Amborella *with respect to the other genomes, with almost no large gene families and a small number of moderate-sized one.

Another question arises because of the great heterogeneity of methods used over more than a decade of genome sequencing, particularly with regard to gene annotation. Most pertinent is the attention paid to identify gene families that are in fact families of transposons. And indeed, the annotation of the recently sequenced *Amborella *genome zealously pursued the identification and exclusion of such families from the set of bonafide gene families. Nevertheless, while this may have ensured a deficit of large families in the data from this genome, it could not account for the observed deficit in families with 8 to 27 genes.

Is the *Amborella *pattern phylogenetically significant? Most of the 43 other genomes are core eudicots, but there is a good number of Poaceae and other monocots, as well as the basal eudicot *Nelumbo*, and these all share the same pattern as the core eudicots. Sequenced genomes of other land plants, like *Selaginella *and *Pinus taeda *are not included in our analysis, and preliminary analyses show other, inconsistent, differences in family size distribution from the angiosperms, but no dearth of large gene families. There is thus no evidence that *Amborella *conserves some ancestral, pre-angiosperm pattern of gene family sizes, but this will question will require further genomic data to settle. A similar question, whether *Amborella *represents a pre-core eudicot pattern among angiosperms, will also require further data from other early branching plants, but already we know that *Nelumbo *as well as the monocots, all have the typical pattern. Another factor may lie in the fact that *Amborella *is the only genome to have escaped whole genome duplication since the origins of the angiosperms; this may also be associated with a lesser tendency to amplify and diversify gene families. Finally, the paucity of large families in *Amborella *may be an acquired feature, and not a conserved one. The current restricted ecological range of this plant may reflect a long history of isolation, of small populations, and little advantage to genetic innovation.

## Competing interests

The authors declare that they have no competing interests.

## Authors' contributions

All authors participated in the research, wrote the paper, read and approved the manuscript.

## Declarations

The publication charges for this article were funded by the Canada Research Chair in Mathematical Genomics, and by the University of Ottawa.

This article has been published as part of *BMC Genomics *Volume 15 Supplement 6, 2014: Proceedings of the Twelfth Annual Research in Computational Molecular Biology (RECOMB) Satellite Workshop on Comparative Genomics. The full contents of the supplement are available online at http://www.biomedcentral.com/bmcgenomics/supplements/15/S6.
